# Identification of Mood Disorders in Self‐Reported Versus Health Administrative Data

**DOI:** 10.1002/brb3.70126

**Published:** 2024-11-07

**Authors:** Irène Dohouin, Maude Laberge, Anaïs Lacasse, Thomas G. Poder, TorSaDE Cohort working group

**Affiliations:** ^1^ School of public health University of Montreal Montreal Quebec Canada; ^2^ Research center of the IUSMM CIUSSS de l'Est de l'île de Montréal Montreal Quebec Canada; ^3^ Faculty of Medicine University Laval Québec Canada; ^4^ Vitam Centre de recherche en santé durable, Université Laval Québec Canada; ^5^ Centre de recherche du CHU de Québec, Université Laval Québec Canada; ^6^ Department of Health Sciences Université du Québec en Abitibi‐Témiscamingue Rouyn‐Noranda Quebec Canada

**Keywords:** health administrative data, linked data, mood disorder, self‐reported data, survey

## Abstract

**Background:**

Producing relevant knowledge on the prevalence of mood disorders (MDs) requires a clear identification of people living with the condition. Analyzing this multifaceted disease from the perspective of health administrative data and population‐based surveys could contribute to document inconsistencies between these data sources and highlight the strengths and limitations of each methodological approaches.

**Objectives:**

The aim of this study was to estimate the prevalence of MD disease, assess concordance of MD patterns in population‐based surveys versus health administrative data, and investigate statistical differences in characteristics between individuals presenting the disease in each data sources.

**Methods:**

This study used the Care Trajectories—Enriched Data (TorSaDE) cohort. The TorSaDE cohort is built by merging five waves of the Canadian Community Health Survey (CCHS) with health administrative data of the province of Quebec, Canada. The sample includes individuals who participated in at least one round of CCHS and for whom evidence of use of health services in the year of CCHS completion and the year before were present in health administrative data. The cohort was split into four groups based on the presence and absence of MD in self‐reported versus health administrative data. Groups' characteristics were compared using chi‐square tests and ANOVA.

**Results:**

The study cohort was composed of 96,079 individuals, of which 10,418 (10.8%) had MD, regardless of the data sources. Self‐reported prevalence of MD was 6.03%, while the prevalence from health administrative data was about 7.79%. Estimates showed a low level of concordance between the two measures, as only 27.4% of people presenting this medical condition were identified in both data sources. Furthermore, individuals identified with MD only in survey data had poorer socioeconomic outcomes but better health outcomes than those from the concordant group (i.e., identified in both data sources). In addition, people presenting MD in health administrative data only had better socioeconomic and health outcomes than those who reported MD diagnosis only in survey data.

**Conclusion:**

Findings suggest that each measure capture different specific subpopulations. Estimates obtained from each source should thus be contextualized and interpreted with caution.

## Graphical abstract

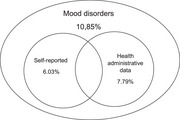

## 1 Introduction

Mood and anxiety disorders are the most common groups of mental disorders worldwide, with mood disorders (MDs), such as major depressive, dysthymic, or bipolar disorders, affecting 4.2% of the global population in 2018 (James et al. 2018). It is estimated that about one‐fifth of Americans suffer from MDs sometime in their life (Kessler et al. 2005). Many studies established MDs to have negative effects on health‐related quality of life (HRQoL) with a strong association with higher suicide rates (Latalova, Kamaradova, and Prasko 2014). In Canada, 3.5 million individuals or 12.6% of the country's population, met the criteria for a MDs during their lifetime, with major depressive episodes accounting for 91% of cases (Pearson, Janz, and Ali 2013). Notably, MD have been identified as the sixth leading contributor to years lived with disability in Canadian settings (Salas‐Wright, Kagotho, and Vaughn 2014). MD have both economic and social costs for the society because of the loss of work productivity experienced by people living with the condition and the high health services costs associated (Simon 2003).

Despite the large number of studies aiming to assess MDs and the profile of individuals diagnosed with this condition, assessment of the real prevalence of MDs is still challenging (O'Donnell et al. 2016). This challenge is mostly associated with the nature and quality of available datasets. Indeed, in this field of study, researchers rely on various data sources, among which health administrative databases and population‐based surveys are the most used. On one hand, health administrative databases offer a unique framework to analyze health conditions for a large population. On the other hand, these data sources, being a by‐product of claims for physicians' reimbursement in a fee‐for‐service billing system, require case‐finding algorithms based on diagnostic codes that are not always valid. Also, they very often do not contain socioeconomic variables (e.g., income, education, work status) that are relevant for the analysis of health profiles (Vanasse et al. 2021). Although health administrative data sources provide information on the real health state from a clinical perspective, they inform only individuals who use health services and seek treatment from health professionals. Yet, it has been shown that a high proportion of people presenting symptoms of MDs do not seek treatment for many reasons, particularly fear of stigma (Lazowski et al.^2012^) and low mental health literacy (Wang et al. 2007; Wang and Lai 2008). Population‐based surveys such as the Canadian Community Health Surveys (CCHS) administrated by Statistics Canada represent alternative data sources frequently used. The CCHS asks participants to self‐report various socioeconomic and health variables, including their health conditions. However, these data sources present some shortcomings, notably the accuracy of self‐reported health problems (survivor and recall bias) as well as barriers that may impede the participation of certain vulnerable populations (Association for Canadian Studies 2010).

Accurate population‐based prevalence estimates of MD are crucial as they help to understand the social burden in the population while directly informing health service planning and resource allocation (Kirkbride 2015). However, little research has been conducted to access and compare the prevalence estimates of MD from the perspective of population surveys and administrative health data. While Edwards et al. (2020) and O'Donnell et al. (2016) have addressed a similar topic at the national level for Canada and for the province of Ontario, respectively, no case study has been documented for the province of Quebec. Also, both studies mentioned above focused on mental disorders (mood and/or anxiety disorders) without distinguishing between them. Results obtained at the national level, even informative, may not be relevant within provinces, especially in Quebec, for various reasons. First, Quebec's population is different from other Canadian provinces. Indeed, it is the only Canadian province that has a predominantly French‐speaking population; and there has been documented a large heterogeneity in mental health outcomes between provinces in Canada (Palay et al. 2019). Second, health systems are under provincial jurisdiction in Canada and access to mental health resources vary widely between provinces. It is, therefore, important to investigate and understand patterns of MD in the specific context of Quebec.

This paper aims to assess whether estimates of self‐reported diagnosis of MD from population‐based survey data are concordant with estimates of clinical diagnosis of these disorders obtained from health administrative data in Quebec; and analyze sociodemographic, and health characteristics associated with potential discrepancies between estimates obtained from these data sources. This will allow us to establish a comparison between a survey‐derived prevalence and an administrative prevalence of MD in Quebec.

## 2 Methods

This study used data from the Care Trajectories—Enriched Data (TorSaDE) cohort (Vanasse et al. 2021). This is a retrospective study that combines five rounds of the CCHS (2007–2008, 2009–2010, 2011–2012, 2013–2014, 2015–2016) conducted by Statistics Canada, with health administrative databases from the Régie de l'assurance maladie du Québec (RAMQ). The RAMQ provides universal health insurance coverage to all residents in the province of Quebec, Canada. The CCHS and RAMQ data were linked under the supervision of the Institut de la Statistique du Québec (ISQ). Access to de‐identified TorSaDE Cohort data was possible through the ISQ secure virtual server (data holder). A detailed description of the TorSaDE Cohort is available elsewhere (Vanasse et al. 2021). Ethical authorizations were obtained from the *Commission d'accès à l'information du Québec* (#1013990) and relevant university Research Ethics Boards (UQAT: #2018‐02; CIUSSSE‐CHUS: #2017‐1504). All participants agreed their data from CCHS to be linked with their health administrative data from the RAMQ.

The TorSaDE cohort includes 102,148 individuals who completed 103,241 entries (i.e., some participants took part in more than one cycle of CCHS). Our study sample is constituted as follows: (1) we used one record for each participant by considering the most recent entry for individuals with more than one participation; (2) then, we selected participants who have used health services in the civil year of the CCHS completion or in the year before. The choice of this period was arbitrary and aimed to allow consistency in the RAMQ and CCHS data. This selection procedure led to the inclusion of 96,079 individuals; (3) next, we considered only participants for whom information on MD health status was available in CCHS. Finally, 95,978 individuals were included for analysis.

### 2.1 Identifying Individuals With MDs

Using RAMQ data, we identified individuals diagnosed with MD in the year of CCHS completion and the year before. For this purpose, we used classification codes ICD‐9 and ICD‐10. We had to use both versions since ambulatory (i.e., non‐hospital‐based) clinics still use ICD‐9, whereas version 10 is implemented in all hospitals. The codes used in this study are presented in Tables A1 and A2. We considered an individual being diagnosed with MD in RAMQ data if he/she received a clinical diagnosis and health services for depression, bipolar disorder, mania, or dysthymia. According to estimates from RAMQ data, 84.35% of diagnoses were related to depression, 1.38% to bipolar disorder, 10.73% to mania, and 3.54% to dysthymia and other MD. Using CCHS, we identified individuals who reported having received a MD diagnosis and were still suffering from this disease at the time of the survey. Indeed, in CCSH, participants were asked to answer the following question labeled CCC_280: “Do you have a mood disorder such as depression, bipolar disorder, mania, or dysthymia?” This question was included in the chronic conditions module, which focused on conditions diagnosed by a health professional that had lasted or were expected to last 6 months or longer. However, the time frame when they received their MD diagnosis was not specified.

### 2.2 Sociodemographic Characteristics

In this study, various variables were compared across groups. We considered sociodemographic characteristics based on answers provided by respondents in the CCHS. These variables included age, sex at birth, educational attainment, marital status, employment status, living alone, urban versus rural residency, whether the CCHS questionnaire was completed by a third party, individual yearly income, or household yearly income. To construct individual income groups, we followed Dufour, Vedel, and Quesnel‐Vallée (2022) by using a threshold of $20,000, which corresponds approximatively to the mean low‐income cut‐offs for the years of our study.

### 2.3 Health Characteristics

Using health administrative data, we reported the combined Charlson–Elixhauser comorbidity index as defined by Simard, Sirois, and Candas (2018) and computed by the TorSaDE team. We considered the index calculated for 1 year before the date of CCHS completion using ICD‐9 and ICD‐10 code systems. The index was computed following a specific procedure. First, both lists of the 17 Charlson (Charlson et al. 1987) and the 30 Elixhauser (Elixhauser et al. 1998) medical conditions were retrieved from the work of Quan et al. (2005). These lists were adapted to the specific context of Quebec and combined to obtain a broader list of 32 medical conditions. Then, ICD‐9 codes were translated into ICD‐10 codes and vice versa. Discrepancies between ICD‐9 and ICD‐10 were resolved with the assistance of a senior registrar or a medical specialist in the relevant medical field. Comorbidity weights were derived for each health condition by applying the Charlson (CH) (Charlson et al. 1987) derivation methods on the estimated coefficients of a logistic regression predicting death within 30 days of hospital admission. Independent variables in the logistic regression included 5‐year age groups, sex, and a dummy variable for the presence of every medical condition. The CH method rounds up odds ratios > 1.2 to the nearest integer for each statistically significant medical condition with a maximal value of 6. Non‐significant medical conditions receive a weight of 0 (Simard, Sirois, and Candas 2018). A lower score indicates a lower morbidity. We also reported the proportion of MD identified during an episode of hospitalization, the number of cases diagnosed 1 and 2 years after the CCHS completion, the duration of illness (i.e., the number of years since the disease had been identified for the first time in RAMQ data). In the CCHS, we considered the self‐assessment of general as well as the mental health status of the participant (i.e., GEN_01 and GEN_02B in CCHS: “In general, would you say that your (mental) health is: 1 Excellent, 2 Very good, 3 Good, 4 Fair, 5 Bad?”).

### 2.4 Use of Services Computed

Information on the use of health services in the RAMQ database was identified. It included the number of visits to a family physician, the number of hospitalization days, and the average number of monthly medications. This was computed for the year before the completion of the CCHS.

### 2.5 Statistical Analysis

Our analysis built on the work of Edwards et al. (2019), who analyzed the prevalence of mood and anxiety disorders in the province of Ontario and Dufour, Vedel, and Quesnel‐Vallée (2022), who performed a study on major cognitive disorders in a population aged 65 years or more. First, we analyzed MD through self‐reported and health administrative data by computing the joint distribution according to both data sources. Second, we classified each person in our sample into two groups: (1) self‐reported and/or administrative MD; and (2) no MD. The first group contains individuals identified with MD in one of the two data sources and the second group contains all individuals without MD. We investigated sociodemographic and health characteristics associated with these disorders by computing and comparing sociodemographic and health characteristics between the two groups. For each characteristic, we assessed differences by implementing a mean‐comparison test (chi‐square test) between the two groups. When differences were significant, we supplemented this analysis by controlling for age and sex. This was done through a simple linear regression (for continuous variables) and a logistic regression (for categorical variables) where independent variables were MD status, age, and sex.

Finally, we assessed the factors associated with the discordances that were observed between self‐reported (i.e., CCHS) and health administrative data. To do so, we classified each person identified with MD in one of the following groups: (A) MD in self‐reported data only (i.e., individuals identified with MD in CCHS only); (B) MD in health administrative data (i.e., individuals identified with MD in RAMQ data only); (C) MD in self‐reported and health administrative data (i.e., individuals identified with MD in both CCHS and RAMQ data). By doing so, we compared MD in self‐reported versus health administrative data and computed summary statistics over each of these three groups. We chose this analysis option in favor of a classic case identification algorithm validation study (Benchimol et al. 2011) since, in our case, our objective was to compare the two data sources on an equal footing, without establishing a priori reference standard measurement. In fact, neither self‐reported nor health administrative data can be considered a gold standard for MD case identification. We measured the differences between the groups for each characteristic using overall chi‐square tests for categorical variables and overall ANOVAs for continuous variables. We supplemented these tests by accounting for the effect of age and sex at birth (i.e., to provide adjusted *p* values). To do so, we implemented a logistical analysis for categorical variables (i.e., were categorized as binary) and a linear regression model for continuous variables (not provided). When differences between groups were significant, we implemented a post hoc analysis using multiple pairwise analysis for chi‐square test and Tukey test for ANOVA (Lee and Lee 2018). A significant level of *p* < 0.001 for all analyses was considered.

## 3 Results

### 3.1 Summary Statistics Over the Sample and Prevalence of MDs in Self‐Reported Versus Health Administrative Data

The study sample consisted of 96,079 individuals, of whom 101 (0.11%) did not report whether they have MD. Those individuals were excluded from our study. The remaining sample (95,978) was composed of 42,597 men (44.38%) and 53,381 women (55.62%). They were 49.2 (SD = 20.2) years old on average, and 69,245 (72.9%) of them completed high school at least. One half of the sample (50.6%) was committed to a marital union (married or living with a partner). The rate of urban residency was 72%. Also, 66% of individuals earned an annual income of $20,000 or more, and 67% of them had worked in the last 12 months before the survey. Moreover, 88% of participants reported having a good or excellent general health condition and 95% indicated having good or excellent mental health.

Table 1 reports the prevalence of MD in CCHS versus health administrative data. The prevalence of MD in self‐reported data (i.e., CCHS) was 6.03% (*n* = 5792; 95% CI = 5.88%–6.18%). The administrative prevalence was 7.79% (*n* = 7481; 95% CI = 7.63%–7.97%); the total prevalence from both data sources was 10.85% (*n* = 10,418; 95% CI = 10.65%–11.05%). Furthermore, estimates revealed discordances for 7563 people (i.e., 7.88% of the study cohort). Indeed, 4626 (5.13%) individuals who reported not being diagnosed with MD in CCHS were identified with this condition in health administrative data, while 2937 (3.32%) without MD diagnosed in health administrative data self‐reported having this health problem in the CCHS. About 49.3% of individuals who reported suffering from MD in CCHS were also identified with this condition in the health administrative data. By comparison, 38.2% of individuals diagnosed with MD in the health administrative data also reported the same health condition in the CCHS.

**TABLE 1:** Mood disorder in self‐reported versus health administrative data.

	Self‐reported assessment in CCHS survey			
		MD absent	MD present	Total
Health administrative data	MD absent	85,560 (94.87) (96.68)	2937 (50.71) (3.32)	88,497 (92.21)
	MD present	4626 (5.13) (61.84)	2855 (49.29) (38.16)	7481 (**7.79**)
Total		90,186 (93.97)	5,792 (**6.03**)	95,978 (100)

*Note*: Results are presented in each cell in this order: frequency, column percentage, and row percentage. Prevalence according to data source is provided in bold.

Abbreviation: MD, mood disorder.

### 3.2 Characteristics of Individuals

Table 2 compares the sociodemographic and health characteristics of people with a self‐reported and/or administrative MD diagnosis to those with no MD diagnosis. Estimates show statistically significant differences between the two groups for each characteristic considered, even after accounting for age and sex at birth. Specifically, women were more likely to be identified with MD than men, as 66.2% of people identified with MD were women, while this proportion was 54.3% in the group without MD. Also, people with MD were more likely to live alone, less likely to be involved in a marital union, more likely to complete a high school degree, less likely to be active in the last 12 months labor market, more likely to earn less than $20,000. Furthermore, they were more likely to have urban residency and less likely to be born abroad. Regarding health characteristics and compared to people without MD, people identified with MD had a higher comorbidity index (0.4887 ± 0.0283) and were less likely to report good general or health conditions. Also, they had a higher number of hospitalization days (1.6591 ± 0.1703), a higher number of family physician visits (3.875 ± 0.074), and a higher number of monthly medications (9.2618 ± 0.1916). Overall, being identified with MD was associated with lower health outcomes.

**TABLE 2:** Characteristics of overall sample and individuals with MD or not.

	Total population	No MD	MD (Self‐reported and/or HAD)	*p*‐value (*χ* ^2^)	*p‐value* adjusted for age and sex
Total	95,978 (100)	85,560 (89.15)	10,418 (10.85)		
Age (years)	49.190 ± 0.128	49.065 ± 0.138	50.210 ± 0.326	< 0.0001	NA
Female	53,381 (55.62)	46,489 (54.33)	6892 (66.15)	< 0.0001	NA
Living alone	28,158 (29.34)	23,817 (27.84)	4341 41.67)	< 0.0001	< 0.0001
Marital status					
Single/divorced/separated	47,414 (49.42)	41,292 (48.28)	6122 (58.80)	< 0.0001	< 0.0001
Married/living with a partner	48,519 (50.58)	44,230 (51.72)	4289 (41.20)		
Household income					
< 40,000 CAD	35,158 (36.64)	30,077 (35.17)	5081 (48.78)	< 0.0001	< 0.0001
≥ 40,000 CAD	60,790 (63.36)	55,454 (64.83)	5336 (51.22)		
Personal income					
< 20,000 CAD	26,730 (33.94)	23,150 (33.11)	3580 (40.54)	< 0.0001	< 0.0001
≥ 20,000 CAD	52,019 (66.06)	46,769 (66.89)	5250 (59.46)		
Education					
High school not completed	25,777 (27.13)	23,274 (27.48)	2503 (24.25)	< 0.0001	< 0.0001
High school at least completed	69,245 (72.87)	61,425 (72.52)	7820 (75.75)		
Employment					
Didn't work last 12 months	26,692 (32.42)	23,124 (31.73)	3568 (37.70)	< 0.0001	< 0.0001
Worked last 12 months	55,647 (67.58)	49,751 (68.27)	5896 (62.30)		
Residency					
Rural	26,.745 (27.87)	24,122(28.19)	2623 (25.18)	< 0.0001	< 0.0001
Urban	69,233 (72.13)	61,438 (71.81)	7795 (74.82)		
Birth country					
Outside Canada	7316 (7.62)	6657 (7.78)	659 (6.33)	< 0.0001	< 0.0001
Canada	88,662 (92.38)	78,903 (92.22)	9759 (93.67)		
Survey completed by a third party	11,459 (11.95)	10,448 (12.22)	1011 (9.710)	< 0.0001	< 0.0001
Comorbidity index (based on ICD‐9)	0.306 ± 0.007	0.283 ± 0.008	0.489 ± 0.028	< 0.0001	< 0.0001
Comorbidity index (based on ICD‐1O)	0.335 ± 0.008	0.314 ± 0.008	0.500 ± 0.030	< 0.0001	< 0.0001
MD identified 1 year following the date index	1399 (1.68)	1185 (1.47)	214 (7.50)	< 0.0001	< 0.0001
MD identified 2 years following the date index	2431 (5.07)	2085 (4.50)	346.000 (20.68)	< 0.0001	< 0.0001
Years since Dx RAMQ	6.743 ± 0.131	6.743 ± 0.131	NA	NA	NA
MD identified during hospitalization	842 (28.74)	NA	842 (28.74)	NA	NA
Health					
Poor/fair	11,635 (12.13)	8684 (10.16)	2951 (28.35)		
Good/excellent	84,276 (87.87)	76,819 (89.84)	7.457 (71.65)	< 0.0001	< 0.0001
Mental health					
Poor/fair	4231 (4.49)	2000 (2.38)	2231 (22.02)		
Good/excellent	89,911 (95.51)	82,010 (97.62)	7901 (77.98)	< 0.0001	< 0.0001
Past hospitalization days	0.692 ± 0.031	0.575 ± 0.027	1.659 ± 0.170	< 0.0001	< 0.0001
Past family physician visits	2.329 ± 0.018	2.140 ± 0.018	3.875 ± 0.074	< 0.0001	< 0.0001
Average number of monthly medications	6.655 ± 0.057	6.292 ± 0.058	9.262 ± 0.192	< 0.0001	< 0.0001

Abbreviations: CAD, Canadian dollar; HAD, health administrative data; MD, mood disorder; RAMQ, Régie de l'assurance maladie du Québec.

### 3.3 Comparative Analysis

Table 3 reports the characteristics of individuals diagnosed with MD distributed according to the three groups defined above (A) MD in self‐reported data only; (B) MD in health administrative data only; and (C) MD in self‐reported and health administrative data. Since the ideal setting is to have perfect concordance between the two data sources so that both display the same pattern, we called the first two groups, *discordant groups* and the last one, *the concordant group*. Results show high discordance between data sources. Indeed, among individuals presenting MD, 27.40% were identified in both databases: 28.19% in self‐reported data (CCHS) only and 44.40% in health administrative data only. Also, overall ANOVAs and overall chi‐square tests revealed statistically significant differences between groups for each characteristic even after accounting for age and sex, except for residential areas and third‐party completion of the survey, as all the *p* values associated with these comparison tests were below the threshold of 0.001. Concerning pairwise comparison, we compared the two discordant groups to the concordant group.

**TABLE 3:** Sociodemographic and health characteristics of individuals presenting MD in survey versus health administrative data.

	Whole subsample	Group A: Self‐reported MD only	Group B: HAD MD only	Group C: Self‐reported and HAD MD	*p*‐value (*χ* ^2^)	*p‐value* adjusted for age and sex	Pairwise comparisons
Total	10,418 (100)	2937 (28.19)	4626 (44.40)	2855 (27.40)			
Age (years)	50.210 ± 0.326	51.010 ± 0.629	50.682 ± 0.509	48.625 ± 0.561	< 0.0001	NA	
Female	6892 (66.15)	1829 (62.27)	3143 (67.94)	1920 (67.25)	< 0.0001	NA	A#B, A#C
Living alone	4341 (41.67)	1240 (42.22)	1812(39.17)	1289 (45.15)	< 0.0001	< 0.0001	A#B, A#C B#C
Marital status							
Single/divorced/separated	6122 (58.80)	1760 (59.97)	2547 (55.11)	1815 (63.59)	< 0.0001	< 0.0001	A#B, A#C B#C
Married/living with a partner	4289 (41.20)	1175 (40.03)	2075 (44.89)	1039 (36.41)			
Household income							
< 40,000 CAD	5081 (48.78)	1591 (54.19)	1999 (43.21)	1491 (52.22)	< 0.0001	< 0.0001	A#B B#C
≥ 40,000 CAD	5336 (51.22)	1345 (45.81)	2627 (56.79)	1364 (47.78)			
Personal income							
< 20,000 CAD	3580 (40.54)	1207 (49.81)	1282 (32.56)	1091 (44.17)	< 0.0001	< 0.0001	A#B, A#C B#C
≥ 20,000 CAD	5250 (59.46)	1216 (50.19)	2655 (67.44)	1379 (55.83)			
Education							
High school not completed	2503 (24.25)	895 (30.84)	995 (21.69)	613 (21.63)			
High school at least completed	7820 (75.75)	2007 (69.16)	3592 (78.31)	2221 (78.37)	< 0.0001	< 0.0001	A#B, A#C
Employment							
Didn't work last 12 months	3568 (37.70)	1146 (44.06)	1296 (31.19)	1126 (41.58)	< 0.0001	< 0.0001	A#B B#C
Worked last 12 months	5896 (62.30)	1455 (55.94)	2859 (68.81)	1582 (58.42)			
Residency							
Rural	2623 (25.18)	775 (26.39)	1164 (25.16)	684 (23.96)	0.103	NA	NA
Urban	7795 (74.82)	2162 (73.61)	3462 (74.84)	2171 (76.04)			
Birth country							
Outside Canada	659 (6.33)	211 (7.18)	303 (6.55)	145 (5.08)	0.003	0.0041	A#C B#C
Canada	9759 (93.67)	2726 (92.82)	4323 (93.45)	2710(94.92)			
Survey completed by a third party	1011 (9.71)	291 (9.91)	459 (9.93)	261 (9.14)	0.487	NA	NA
Comorbidity index (based on ICD‐9)	0.489 ± 0.028	0.434 ± 0.052	0.520 ± 0.045	0.495 ± 0.051	< 0.0001	< 0.0001	A#B, A#C
Comorbidity index (based on ICD‐10)	0.500 ± 0.030	0.432 ± 0.053	0.550 ± 0.047	0.487 ± 0.052	< 0.0001	< 0.0001	A#B, A#C
MD identified 1 year following the date index	214 (7.50)	214 (7.50)	NA	NA	NA	NA	
MD identified 2 years following the date index	346 (20.68)	346 (20.68)	NA	NA	NA	NA	
Years since Dx RAMQ	6.743 ± 0.131	NA	5.743 ± 0.159	8.363 ± 0.214	NA		
MD identified during hospitalization	842 (28.74)	NA	380 (21.62)	462(39.42)	< 0.0001	< 0.0001	B#C
Health							
Poor/fair	2951 (28.35)	1059 (36.11)	832 (18.00)	1060 (37.15)	< 0.0001	< 0.0001	A#B, A#C B#C
Good/excellent	7457 (71.65)	1874 (63.89)	3790 (82.00)	1793 (62.85)			
Mental health							
Poor/fair	2231 (22.02)	797 (28.64)	366 (8.03)	1068 (38.24)	< 0.0001	< 0.0001	A#B, A#C B#C
Good/excellent	7901 (77.98)	1986 (71.36)	4190 (91.97)	1725 (61.76)			
Past hospitalization days	1.659 ± 0.170	0.952 ± 0.248	1.607 ± 0.236	2.472 ± 0.416	< 0.0001	< 0.0001	A#B, A#C B#C
Past family physician visits	3.875 ± 0.074	3.108 ± 0.115	3.906 ± 0.111	4.615 ± 0.158	< 0.0001	< 0.0001	A#B, A#C B#C
Average number of monthly medications	9.262 ± 0.192	8.954 ± 0.331	8.971 ± 0.302	10.038 ± 0.372	0.282	NA	

Abbreviations: CAD, Canadian dollar; HAD, health administrative data; MD, mood disorder; RAMQ, Régie de l'assurance maladie du Québec.

On the one hand, relative to individuals identified with MD in both data sources, those identified in CCHS only had higher rate of marital union (40.3% vs. 36.41%), were less likely to complete high school degree (69.19% vs. 78.37%), were less likely to earn annually $20,000 or more (50.19% vs. 55.83%), and were more likely to be born abroad (7.2% vs. 5.1%). Also, they had a lower comorbidity index (0.434 ± 0.053 vs. 0.495 ± 0.051) and were more likely to report higher perceived mental health outcomes (71.36% vs. 61.76%). Furthermore, they had a lower number of hospitalization days (0.952 ± 0.242 vs. 2.472 ± 0.416) and recorded fewer visits to their family physicians (3.108 ± 0.115 vs. 4.615 ± 0.158). Overall, individuals reporting MD only in CCHS experienced poorer sociodemographic and better health outcomes compared to those from the concordant group.

On the other hand, compared to individuals identified with MD in both data sources, those identified only in administrative data had higher rate of marital union (44.89% vs. 40.3%), showed a higher labor market activity rate over the past 12 months (68.81% vs. 58.42%), were more likely to earn $20,000 or more as annual income (67.44% vs. 55.83%), and were more likely to be born abroad (6.55% vs. 5.1%). Also, they showed a statistically similar comorbidity index (0.520 ± 0.045 vs. 0.495 ± 0.051) and were more likely to report higher perceived mental health outcomes (91.97% vs. 61.76%). Furthermore, they showed fewer duration since the first diagnosis of the disease (5.743 ± 0.159 vs. 8.363 ± 0.214) had a lower number of hospitalization days (1.607 ± 0.236 vs. 2.472 ± 0.416) and fewer visits to their family physicians (3.906 ± 0.111 vs. 4.615 ± 0.158). Overall, individuals reporting MD only in administrative data experienced better sociodemographic and health outcomes compared to those from the concordant group.

Moreover, we analyzed the differences between the two discordant groups. We observed that relatively to people identified with MD only in CCHS, those who received a positive diagnosis only in health administrative data (RAMQ) had higher rate of marital union (44.89% vs. 36.41%), were more likely to complete high school degree (78.31% vs. 69.19%), showed a higher labor market activity rate over the past 12 months (68.81% vs. 55.94%), and were more likely to earn $20,000 or more as annual income (67.44 % vs. 50.19%). Also, they showed a higher comorbidity index (0.520 ± 0.045 vs. 0.434 ± 0.053) and were more likely to report higher perceived mental health outcomes (91.97% vs. 71.36%). Furthermore, they had a higher number of hospitalization days (1.607 ± 0.236 vs. 0.952 ± 0.242) and recorded more visits to their family physicians (3.906 ± 0.111 vs. 3.108 ± 0.115). Overall, individuals reporting MD only in health administrative data experienced better sociodemographic and worse health outcomes compared to those from CCHS only, except for the perceived mental health.

## 4 Discussion

In this study, we investigated the prevalence of MDs and compared the characteristics of people based on their status on MDs in self‐reported and health administrative data in Quebec, Canada. Our findings suggest a low level of concordance between the two data sources, as only 27.4% of people presenting the disease were diagnosed in both data sources. Also, results indicated a prevalence of MDs of 7.79% based on health administrative data and 6.03% using self‐reported data. This result agrees with the work of O'Donnell et al. (2016), who found that the prevalence of mental disorders (mood and/or anxiety disorders) derived from health administrative data were consistently higher than ones derived from population surveys data (i.e., 11.3% vs. 9.4%). A hypothesis suggested by Palin et al. (2012) to explain lower self‐reported rates is that administrative records may capture less severe or subclinical cases compared to survey data. A way to explain this hypothesis is that in the frame of our study, people identified with the disease in administrative records might not report the diagnosis in CCHS data because they faced less severe cases of MDs or had no outward symptoms or signs, which results in a higher prevalence estimates from administrative data compared to survey data. However, in another context, it is also plausible that those diagnosed by a professional and consequently registered in administrative records are individuals with severe cases that cannot be concealed. This is particularly relevant given that people with these illnesses might face social stigma, including from healthcare professionals.

Inconsistencies in the identification of health conditions have been documented in the literature, either for mental disorders or other health conditions. Dufour, Vedel, and Quesnel‐Vallée (2022) analyzed the consistency of identification of major cognitive disorders in self‐reported and health administrative data and showed that 70% of individuals presenting this health problem in administrative data did not report it in household surveys. A study by Jiang et al. (2015) analyzed concordance between self‐reported and health administrative data for chronic diseases. They found a substantial agreement for diabetes, moderate agreement for heart disease, and a fair agreement for depression. Another study by Singh (2009) on US veterans showed that among individuals presenting with a clinical diagnosis of depression, roughly 83% self‐reported the condition in survey data.

Estimates showed systematic differences in socioeconomic and health characteristics of people presenting the health condition in one of the two data sources compared to those without MD in both data sources. Moreover, we found significant differences between the three groups of people identified with MD, except for residency and whether the survey was completed by a third party. Indeed, compared to people presenting the disease in both databases, individuals identified in survey data only had poorer socioeconomic backgrounds, but enjoyed better health outcomes. Compared to the two previous groups, people identified only in administrative records had better socioeconomic backgrounds. These findings suggest that each data source may identify different subpopulations suffering from MD and support the work by Edwards et al. (2020), which led to similar conclusions for the province of Ontario.

Such differences may partially be explained by specific limitations associated with both data sources. Population‐based surveys rely on self‐assessment, which may be particularly problematic for MDs due to issues related to the severity of health conditions, stigma or self‐stigma, and recall bias. Also, identifying the actual prevalence of MDs in administrative data is challenging because of the complexity of the diagnosis process as well as the perceived validity of clinical codes used. Indeed, as for most mental diseases, there is no objective test to assign a diagnosis; hence, a diagnosis is based on the signs and symptoms reported by the patient or his/her relatives, as well as those observed by the physician (Pies 2007), and medical diagnosis procedures may vary from a place to another. Consequently, it is possible that individuals can experience symptoms without meeting standard diagnostic criteria and yet receive a diagnosis (O'Donnell et al. 2016). Moreover, the simultaneous co‐occurrence of other mental diseases, such as anxiety or personality disorders, exacerbates the diagnostic process difficulty (Friborg et al. 2014). In addition, some discrepancies may be due to a lack of access in mental healthcare services.

Overall, this study showed that using only one of these data sources may be insufficient for assessing the prevalence and characterizing population suffering from MD. Consequently, results should be interpreted with caution and decisions about health planning, resource allocation or any critical health decisions should be taken after analyzing the context from the perspective of both measures of MDs.

### 4.1 Strengths and Limitations

First, our study used the TorSaDE cohort, a combination of cross‐sectional patient self‐reported outcomes with health administrative data to identify MDs in the Quebec population. This combination of two complementary datasets offers a unique framework to address the topic in the absence of clinical‐based cohort data. However, this study may suffer from biases associated with the self‐reporting of health problems, as well as limited information on individuals, such as the timing and duration of MD.

Also, our methodology allowed consistency and comparability of estimates from self‐reported and health administrative data since we targeted the same individuals in the two datasets. However, by focusing only on individuals who used health services during the year of CCHS completion and the year before, our study failed to account for individuals who may have symptoms of MDs but do not have any contact with the health system during the reference period. More generally, our methodology did not allow us to take advantage of probability sampling, which is one of the main strengths of CCHS.

Moreover, although some respondents may have participated multiple times in the CCHS, our sample was limited to only one CCHS record per participant, namely, the most recent entry. While this approach has the strength of allowing accuracy in the current prevalence rate of MD, it does not allow us to analyze changes over time.

## 5 Conclusion

MDs are diseases that negatively affect the HRQoL and are strongly associated with higher suicide rates. Evaluating the quality of care to improve the well‐being of the population affected by this condition requires knowledge of their characteristics. This study provided insights in this direction and showed that health administrative data generated a higher prevalence of MDs in the Quebec population. Future research is needed to address the discrepancies observed in this study and for others, as well as to study the implications for researchers and clinicians.

## Author Contributions

**Irène Dohouin**: writing–original draft, methodology, visualization, formal analysis, data curation. **Maude Laberge**: funding acquisition, writing–review and editing, validation. **Anaïs Lacasse**: funding acquisition, validation, writing–review and editing. **Thomas G. Poder**: conceptualization, investigation, funding acquisition, methodology, validation, writing–review and editing, formal analysis, project administration, supervision.

## Conflict of Interest

The authors declare no conflicts of interest.

### Peer Review

The peer review history for this article is available at https://publons.com/publon/10.1002/brb3.70126.

## Data Availability

The data that support the findings of this study are openly available in ISQ at https://statistique.quebec.ca/fr.

## 1

**TABLE A1:** ICD‐10.

Mood [affective] disorders (F30–F39)	Codes
Hypomania	F300
Mania without psychotic symptoms	F301
Mania with psychotic symptoms	F302
Other manic episodes	F308
Manic episode, unspecified	F309
Bipolar affective disorder, current episode hypomanic	F310
Bipolar affective disorder, current episode manic without psychotic symptoms	F311
Bipolar affective disorder, current episode manic with psychotic symptoms	F312
Bipolar affective disorder, current episode mild or moderate depression	F313
Bipolar affective disorder, current episode severe depression without psychotic symptoms	F314
Bipolar affective disorder, current episode severe depression with psychotic symptoms	F315
Bipolar affective disorder, current episode mixed	F316
Bipolar affective disorder, currently in remission	F317
Other bipolar affective disorders	F318
Bipolar affective disorder, unspecified	F319
Mild depressive episode	F320
Moderate depressive episode	F321
Severe depressive episode without psychotic symptoms	F322
Severe depressive episode with psychotic symptoms	F323
Other depressive episodes	F328
Depressive episode, unspecified	F329
Recurrent depressive disorder, current episode mild	F330
Recurrent depressive disorder, current episode moderate	F331
Recurrent depressive disorder, current episode severe without psychotic symptoms	F332
Recurrent depressive disorder, current episode severe with psychotic symptoms	F333
Recurrent depressive disorder, currently in remission	F334
Other recurrent depressive disorders	F338
Recurrent depressive disorder, unspecified	F339
Cyclothymia	F340
Dysthymia	F341
Other persistent mood [affective] disorders	F348
Persistent mood [affective] disorder, unspecified	F349
Other single mood [affective] disorders	F380
Other recurrent mood [affective] disorders	F381
Other specified mood [affective] disorders	F388
Unspecified mood [affective] disorder	F39

**TABLE A2:** ICD‐9.

Name of diseases	Codes
Depressive disorder, not elsewhere classified, except 3004‐3080‐3090‐3091‐3011‐296‐2980‐3131‐3094	311
Neurotic depression, except 311‐2980‐2961‐3090	3004
Depressive disorder, not elsewhere classified—unspecified	3119
Affective psychoses, except 3004‐2981‐2980	296
Manic‐depressive psychosis, manic type, except 2962	2960
Manic‐depressive psychosis, depressed type, except 311–2963	2961
Manic‐depressive psychosis, circular type but currently manic, except 2968	2962
Manic‐depressive psychosis, circular type but currently depressed, except 2968	2963
Manic‐depressive psychosis, circular type, mixed	2964
Manic‐depressive psychosis, circular type, current condition not specified	2965
Manic‐depressive psychosis, other, and unspecified	2966
Other affective psychoses, except 298	2968
Affective psychoses—unspecified	2969
